# Cancer-Associated Fibroblast Heterogeneity and Extracellular Matrix Remodeling as Orchestrators of Drug Resistance in Upper Gastrointestinal Cancers: Insights from Spatial Multi-Omics and Therapeutic Implications

**DOI:** 10.3390/cancers18142358

**Published:** 2026-07-22

**Authors:** Yasamin Mirzabeigi, Joe Youssef, Jeffrey Gonzalez, Thais Martinez, Rima Avellan, Andres Wong, Miguel Perez, Luis Lorenzo Carvajal, Wassim Abou-Kheir, Hisham F. Bahmad

**Affiliations:** 1 Department of Pathology and Laboratory Medicine, University of Miami Miller School of Medicine, Miami, FL 33136, USA; yasamin.mirzabeigi@miami.edu; 2 Department of Anatomy, Cell Biology, and Physiological Sciences, Faculty of Medicine, American University of Beirut, Beirut 1107, Lebanon; jmy06@mail.aub.edu; 3 Herbert Wertheim College of Medicine, Florida International University, Miami, FL 33199, USA; jgonz1074@med.fiu.edu (J.G.); tmart148@med.fiu.edu (T.M.); ravel001@med.fiu.edu (R.A.); awong075@med.fiu.edu (A.W.); mpere943@med.fiu.edu (M.P.); llore049@med.fiu.edu (L.L.C.)

**Keywords:** cancer-associated fibroblasts, extracellular matrix, tumor microenvironment, drug resistance, upper gastrointestinal cancers, spatial transcriptomics

## Abstract

Upper gastrointestinal (GI) cancers, including esophageal, gastric, and pancreatic cancers, remain among the most lethal malignancies worldwide, mainly because they resist nearly every therapeutic modality, from platinum-based chemotherapy, and anti-HER2 and anti-VEGF agents, to immune checkpoint inhibitors. Although tumor cell-intrinsic resistance is well characterized, an increasing share of treatment failure traces to the tumor microenvironment (TME), where cancer-associated fibroblasts (CAFs) and the extracellular matrix (ECM) act not as passive stroma but as active orchestrators of resistance. Here we argue that the functional heterogeneity of CAFs, spanning myofibroblastic (myCAF), inflammatory (iCAF), and antigen-presenting (apCAF) subtypes, and the desmoplastic ECM they construct converge on a small number of shared resistance programs. Those include paracrine signaling, metabolic reprogramming, extracellular vesicle (EV) transfer, and biomechanical remodeling that together drive chemoresistance, targeted therapy evasion, and immune exclusion. Emerging spatial multi-omics now resolves these programs to define niches within upper GI tumors, reframing resistance as a spatially organized property of the tissue rather than the tumor cell alone. We bring this evidence together and evaluate strategies aimed at CAF reprogramming and ECM normalization, arguing that spatially resolved targeting of the stroma represents a tractable path to overcoming resistance in these refractory cancers.

## Simple Summary

Cancers of the esophagus, stomach, and pancreas are among the deadliest, in part because they often stop responding to chemotherapy or targeted and immune therapies. Most research has focused on the cancer cells themselves, but the tissue surrounding a tumor also shapes how well the treatment works. This review focuses on two parts of that surrounding tissue: support cells called cancer-associated fibroblasts (CAFs) and the dense scaffold of proteins they build around the tumor, the extracellular matrix (ECM). We describe how different types of these support cells, and the scaffolds they create, help tumors survive treatment by blocking drug delivery, hiding the tumor from the immune system, and altering its behavior. We also discuss new technologies that reveal exactly where these resistance processes occur within a tumor. By bringing this knowledge together, we hope to point toward treatments that target the tumor’s surroundings and help patients respond better to existing therapies.

## 1. Introduction

Upper gastrointestinal (GI) malignancies, including esophageal, gastric, and pancreatic cancers, remain among the most lethal cancers worldwide, largely due to late-stage presentation and resistance to available treatment modalities. Globally, in 2022, gastric cancer accounted for 968,350 new cases and 659,853 deaths [1], while esophageal cancer contributed to over 510,000 new cases and approximately 445,000 deaths [2]. Pancreatic cancer, with an estimated 510,566 new cases and 467,005 deaths, is particularly notable for its high mortality-to-incidence ratio [1]. These malignancies are consistently associated with poor survival outcomes, with late-stage diagnosis representing a major contributing factor. The absence of effective population-based screening further contributes to delayed detection and poor prognosis. Despite advances in treatment, five-year relative survival rates in the United States remain low, with approximately 13% for pancreatic cancer and 22% for esophageal cancer [3].

Poor outcomes among upper GI cancer patients are largely due to the development of resistance across multiple therapeutic modalities. Platinum-based chemotherapy continues to serve as a cornerstone of treatment in these tumors. At the molecular level, several mechanisms contribute to platinum resistance, including upregulation of L-type amino acid transporter 1 (LAT1), which promotes tumor cell survival through activation of the mTOR pathway [4], the activation of the interleukin-6 (IL-6)/signal transducer and activator of transcription 3 (STAT3) pathway [5], the delay in caspase 3/7 activation, and the weaker induction of pro-apoptotic genes [6].

Resistance also remains a major limitation of anti-HER2-targeted therapies in upper GI cancers. Park et al. demonstrated that trastuzumab-resistant gastric cancer cells can retain HER2 expression while activating alternative signaling pathways, allowing proliferation despite therapy [7]. This highlights that resistance may occur despite maintained target expression, driven instead by compensatory signaling mechanisms. Clinically, this is reflected by the observation that approximately half of the patients with HER2-positive gastric and gastroesophageal cancers exhibit primary or early acquired resistance, resulting in limited overall benefit [8,9].

Anti-angiogenic therapy has also demonstrated clinical benefit in upper GI cancers [10,11]. In the phase III RAINBOW trial, the addition of ramucirumab, a VEGFR-2 antagonist, to paclitaxel significantly improved overall survival in patients with previously treated advanced gastric adenocarcinoma [12]. However, the magnitude of benefit remains limited as tumors can bypass VEGF inhibition through activation of alternative pro-angiogenic pathways, including fibroblast growth factor (FGF) and platelet-derived growth factor (PDGF) signaling, adopt a more invasive phenotype under hypoxic conditions, and enhance pericyte-mediated vascular protection [13]. Additionally, VEGF-independent mechanisms such as vessel co-option and vasculogenic mimicry further contribute to therapeutic escape [14].

Immune checkpoint inhibitors (ICIs) targeting the programmed death 1 (PD-1)/programmed death-ligand 1 (PD-L1) axis have demonstrated clinical efficacy in subsets of patients with esophageal and gastric cancers. In the CheckMate 649 trial, the addition of nivolumab (fully human IgG4 monoclonal antibody targeting PD-1) to chemotherapy improved overall survival at 5 years of follow-up in patients with advanced gastric, gastroesophageal junction, and esophageal adenocarcinoma with PD-L1 combined positive score (CPS) ≥5 (HR 0.71, 95% CI 0.61–0.81), with 5-year OS rates of 16% versus 6% [15]. Nonetheless, median survival remains limited, and only a subset of patients showed sustained benefit, highlighting resistance to immune checkpoint inhibition.

All the prior examples illustrate a key biological and clinical challenge, where upper GI cancers frequently fail to respond to major treatment modalities or initially respond but subsequently develop resistance. Indeed, treatment resistance is driven not only by tumor cells but also by the tumor microenvironment (TME) and its components, as well as by interactions between the tumor and the TME. The TME comprises stromal fibroblasts, extracellular matrix (ECM), endothelial cells, and various signaling factors, all of which regulate drug penetration, immune infiltration, and tumor cell plasticity. In upper GI cancers, dysregulated ECM composition is closely associated with increased tumor aggressiveness, poor clinical outcomes, and resistance to therapy, suggesting that the ECM is not merely structural but functionally involved in disease progression [16,17].

Among the various TME components, cancer-associated fibroblasts (CAFs) are particularly abundant and functionally diverse, playing a central role in orchestrating tumor behavior. In stroma-rich tumors such as pancreatic ductal adenocarcinoma (PDAC), the ECM is highly desmoplastic and stiff due to the activation of CAFs [18]. Increased ECM stiffness not only serves as a physical barrier to drug delivery but also alters intracellular signaling pathways, promoting chemoresistance [19]. In gastric cancer, CAFs expressing calponin-1 (CNN1) have been shown to increase ECM stiffness by activating the Rho-associated protein kinase 1 (ROCK1)/myosin light chain (MLC) pathway, thereby promoting resistance to 5-fluorouracil [20]. This provides a mechanistic example of how CAF-driven ECM remodeling directly contributes to chemotherapy resistance. Additionally, collagen-rich ECM has been shown to promote immune evasion through mechanosensory signaling (such as integrin pathways) that upregulates the metabolic enzyme Interleukin-4 Induced 1 (IL4I1) [21].

Despite incremental advances in chemotherapy, anti-HER2, anti-angiogenic, and immune checkpoint strategies, durable responses in upper GI cancers remain the exception rather than the rule, with resistance emerging as a recurring barrier across many therapeutic modalities. While esophageal, gastric, and pancreatic cancers are anatomically distinct and exhibit divergent baseline biology, they share a common reliance on stromal remodeling to evade current therapeutic strategies. Indeed, PDAC is characterized by an extreme desmoplastic architecture driven predominantly by activated pancreatic stellate cells, which contrasts with the luminal, layered microenvironments of gastric and esophageal tumors [22]. However, despite these differences in cellular origins and structural foundations, all three malignancies converge upon a shared stromal logic: the deployment of CAFs and dense ECM networks to establish biophysical barriers and orchestrate immune exclusion, leading to regional therapeutic resistance.

**Figure 1:**
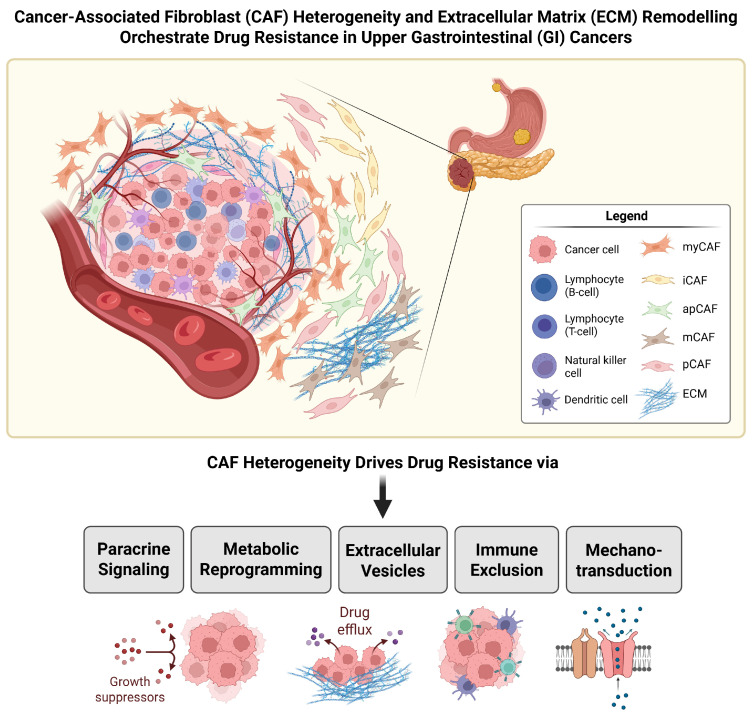
Cancer-associated fibroblast (CAF) heterogeneity and extracellular matrix (ECM) remodeling orchestrate drug resistance in upper gastrointestinal (GI) cancers. Schematic of the tumor microenvironment (TME) in upper GI cancers (gastric, esophageal and pancreatic), showing a vascularized tumor nest of cancer cells infiltrated by immune populations (B and T lymphocytes, natural killer cells, dendritic cells) and surrounded by a heterogeneous, collagen-rich ECM. Multiple CAF subtypes populate the stroma: myofibroblastic (myCAF), inflammatory (iCAF), antigen-presenting (apCAF), matrix (mCAF), and proliferative (pCAF) CAFs, defined as indicated in the legend. CAF heterogeneity promotes drug resistance through five convergent mechanisms (bottom): paracrine signaling, metabolic reprogramming, transfer of extracellular vesicles, immune exclusion, and mechanotransduction. Created in BioRender. Bahmad, H. (2026) https://BioRender.com/oomd49b.

The evidence reviewed here points to a unifying theme where treatment failure is not dictated by tumor cells in isolation but is actively shaped by the TME, in which CAFs and the ECM function as dynamic, heterogeneous regulators of drug penetration, immune infiltration, and tumor cell plasticity. Yet the functional diversity of CAF subtypes and the spatial organization of ECM remodeling remain incompletely understood, in part because conventional bulk analyses obscure the cellular and architectural context in which these interactions occur. In this review, we draw on spatial multi-omics to examine CAF heterogeneity and ECM dynamics as central orchestrators of drug resistance in upper GI malignancies, and we discuss how a deeper, spatially resolved understanding of the TME may inform predictive biomarkers and microenvironment-directed therapeutic approaches (Figure 1).

## 2. Cancer-Associated Fibroblast (CAF) Biology in Upper GI Cancers

### 2.1. Origins and Activation

Cancer-associated fibroblasts (CAFs) are a heterogeneous subpopulation of tumor stroma derived from various cellular precursors. Resident fibroblasts are widely regarded as the predominant source of CAFs, transdifferentiating in response to tumor-secreted factors into activated myofibroblastic cells expressing α-smooth muscle actin (α-SMA; encoded by *ACTA2*) and collagen type I α1 chain (COL1A1) [23]. Other lineages contribute in a context-dependent manner. In murine models of gastric cancer, bone marrow-derived mesenchymal stem cells account for roughly 20% of the intratumoral CAF pool [24], while CD34+/CD45+/Collagen I+ fibrocytes are recruited to the gastric stroma through the stromal cell-derived factor-1 (SDF-1)/CXCR4 axis [25,26] and subsequently differentiate into α-SMA+ myofibroblasts under transforming growth factor β (TGF-β), driving tumor growth in vivo [25]. Endothelial cells represent a further source, generating as much as 40% of the CAF population in some cancer models through endothelial-to-mesenchymal transition (EndMT) [27,28]. Other sources include adipocytes, pericytes, stellate cells, and epithelial cells undergoing epithelial-to-mesenchymal transition (EMT) [29]. A different origin pathway has also been described for antigen-presenting CAFs (apCAFs) in PDAC, which derive from mesothelial cells via mesothelial-to-mesenchymal transition (MMT) driven by paracrine interleukin-1 (IL-1) and TGF-β signaling from tumor cells [30].

### 2.2. Key CAF Subtypes

Three CAFs were initially defined using cross-species single-cell RNA sequencing (scRNA-seq) of human and mouse PDAC samples: myofibroblastic CAFs (myCAFs), inflammatory CAFs (iCAFs), and antigen-presenting CAFs (apCAFs) [31].

The myCAF population expresses ACTA2 (αSMA), TAGLN, THY1, IGFBP3, and COL12A1 and is regulated by the TGF-β pathway to generate a desmoplastic phenotype [31,32,33]. In organoid co-cultures of PDAC, myCAFs localize in direct contact with cancer cells, while iCAFs are located more distally [32,33].

iCAFs are localized distally to tumor cells and express CXCL12, CXCL1, CCL7, and IL6 in PDAC [31,33]. The induction of these cells begins when tumor cells activate the NF-κB and JAK-STAT3 pathways via the secretion of IL-1α; TGF-β inhibits IL1R1, thereby blocking JAK/STAT signaling and establishing an antagonistic regulatory network between iCAF and myCAF populations [32,33,34]. In gastric cancer, iCAFs are enriched in diffuse-type cancers, secreting CXCL1/2/5/6, IL-6, IL-11, and matrix metalloproteinase (MMP)-1/3/10, and play a crucial role in promoting immunosuppression and tumor invasion [35,36]. In esophageal SCC, CAF subpopulations have been identified by scRNA-seq, including iCAFs and myCAFs with distinct marker profiles [37,38], while CXCL1 has been shown to mediate iCAF formation via the CXCR2-pSTAT3 activation pathway in these tumors [39].

apCAFs are characterized by the expression of MHC class II molecules (HLA-DRA, HLA-DRB1, CD74) and can activate CD4+ T cells [31]. In PDAC, apCAFs derived from mesothelial cells ligate naïve CD4+ T cells in an antigen-specific fashion, leading to regulatory T cell (FoxP3+) differentiation [30]. In gastric cancer, Song et al. demonstrated that apCAFs characterized by high MHC II expression were significantly enriched in tumors compared to normal gastric tissues, promoted T cell activation and enhanced cytotoxic and proliferative capacities, and were associated with improved responses to immune checkpoint inhibitors across multiple cancer types [40].

In addition to these three canonical CAF subpopulations, high-resolution scRNA-seq in gastric cancer has revealed further CAF populations, including matrix CAFs (mCAFs; POSTN+), proliferative CAFs (pCAFs; TOP2A+), and pericyte-derived populations [41] (Table 1). This CAF-subtypes nomenclature (myCAF, iCAF, apCAF, mCAF, pCAF) represents transcriptional and functional states rather than fixed cell lineages. Fibroblasts transition between these states as determined by the local IL-1α/TGF-β balance [42]. Furthermore, canonical markers such as FAP, α-SMA (ACTA2), and COL1A1 are shared across subtypes at different expression levels rather than being exclusive to any single population [32,33].

As such, three limitations of this classification are worth mentioning. First, since the CAF subtypes are defined by single-cell transcriptional signatures rather than lineage tracing, the same fibroblast can transition between states over the course of tumor progression or treatment [42]. A single spatial niche may therefore contain a continuum of intermediate phenotypes. Second, a given CAF subtype does not have a fixed function across tumor types: apCAFs drive regulatory T-cell differentiation in PDAC but are associated with T-cell activation and improved immune checkpoint inhibitor response in gastric cancer [30,40], showing that subtype identity alone is an incomplete predictor of effect without tumor type and spatial context. Finally, there is an extensive overlap of canonical markers across subtypes (although at different levels of expression) [32,33]. As a result, single-marker immunohistochemistry probably over- or underestimates true subtype prevalence compared to multiplexed spatial or single-cell approaches.

### 2.3. Spatial Localization Within Upper GI Tumors

Spatial transcriptomics and imaging mass cytometry analysis of 24 PDAC samples (13 poor prognosis and 11 good prognosis) revealed a distinction between two spatial compartments of the stroma. One is the tumor-proximal compartment with a myCAF transcriptional profile (DKK3+, PDPN+, WNT5A+, MMP11+, INHBA+) and the other is the tumor-distal compartment with an iCAF profile (C3+, SFRP2+, STAT3+, IL6+, THY1+, CD34+) [43].

In gastric cancer, spatial transcriptomics analyses have identified transcriptionally distinct tumor stromal subtypes with varying levels of fibroblast infiltration and immune cell composition [44,45]. Fibrotic subtypes, more common among diffuse-type gastric cancers, display high levels of fibroblast infiltration, EMT-related gene program upregulation, and worse prognosis [44]. Spatial proximity of macrophages with fibroblasts has been associated with upregulation of immune checkpoints and inhibition of CD8+ T cell cytotoxicity [45,46]. A separate study using spatial transcriptomics coupled to scRNA-seq revealed that apCAFs (HLA-DRB1+, CD74+) localize in close proximity to cancer cells, as confirmed by multiplex immunohistochemical staining, with apCAF-malignant cell interactions mediated by the receptor-ligand pairs Midkine (MDK)-Nucleolin (NCL) and MDK-Syndecan-2 (SDC2) [41].

### 2.4. CAF Plasticity and Phenotypic Switching

The phenotype of CAFs is flexible in that competing TME signals determine it. The IL-1α/TGF-β antagonistic pathway is the best-studied example of the switch between iCAF and myCAF phenotypes in PDAC. IL-1α secreted by the tumor cells triggers JAK/STAT3 signaling pathways to maintain the iCAF phenotype, whereas TGF-β reduces the expression of IL1R1 receptors and STAT3 to promote the myCAF phenotype [32,33,34,42,47]. These two cytokines compete such that the ratio of IL-1α and TGF-β in the stroma determines their phenotypic fate [33,47].

Additional epigenetic and metabolic controls also influence CAF behavior. In gastric cancer, CAFs show DNA hypomethylation along with epigenetic alterations that lead to increased WNT5A expression, resulting in myCAF phenotype [48]. Pseudotime analysis of gastric cancer scRNA-seq data has shown that pericytes act as a multipotent progenitor population able to produce iCAFs, mCAFs, and apCAFs based on local signals [41]. The CAF subtype displays differentiation plasticity, transitioning from early normal-like states to inflammatory and myofibroblastic phenotypes [34,48]. While direct evidence of phenotypic switching in CAF populations in upper GI tumors following treatment remains limited, these results indicate that CAF programming is reversible.

## 3. Mechanisms by Which CAF Heterogeneity Drives Drug Resistance

### 3.1. Paracrine Signaling Resistance

Paracrine signaling from CAFs involves multiple receptor tyrosine kinase (RTK) and cytokine pathways that confer drug resistance in upper GI cancers. In gastric cancer, Ding et al. identified hepatocyte growth factor (HGF) as a cytokine predominantly originating from CAFs [49]. CAF-derived HGF promoted proliferation, migration, and invasion of MET-unamplified gastric cancer cells through coordinated activation of the HGF/c-Met/STAT3/Twist1 and IL-6/IL-6R/JAK2/STAT3/Twist1 pathways, with enhanced tumorigenesis and metastasis [49,50].

This HGF/MET axis also mediates resistance to HER2-directed therapy. Chen et al. demonstrated that HGF activation of MET receptors rescued HER2-amplified gastric cancer cells from lapatinib (tyrosine kinase inhibitor [TKI] that reversibly targets both the EGFR and HER2 receptors)-induced growth inhibition by restimulating downstream signaling of the activation of protein kinase B (AKT) and extracellular signal-regulated kinase (ERK) pathways and restoring normal cell-cycle progression. Sensitivity to lapatinib correlated with the degree of HER2 amplification, and the rescue effect was abrogated by the MET inhibitor PHA-665752 or MET knockdown, establishing HGF/MET-mediated resistance as a bypass mechanism to HER2-targeted agents [51]. These findings are verified by Zhang et al., who identified MET, HER3, IGF1R, and INSR as mediators of lapatinib unresponsiveness in HER2-positive gastric cancer through restimulation of shared downstream AKT, ERK, and WNT signaling [52].

The IL-6/STAT3 axis represents another critical paracrine mechanism of resistance. Ham et al. demonstrated that IL-6 functions as a CAF-specific secretory protein mediating crosstalk between gastric cancer cells and CAFs. CAF-derived IL-6 activated the JAK1/STAT3 signaling pathway in gastric cancer cells, conferring chemoresistance that was abrogated by the anti-IL-6 receptor antibody tocilizumab, establishing IL-6 as a targetable mediator of stromal chemoresistance [53,54]. In esophageal SCC, Saito et al. demonstrated that fibroblast supernatants induced cell proliferation and resistance to lapatinib through both HGF/MET and FGF/FGFR signaling pathways. The rescue effect against lapatinib was abolished by co-treatment with the FGFR inhibitor PD-173074, indicating that combined inhibition of these RTK pathways may hold therapeutic promise [55].

### 3.2. Metabolic Reprogramming

Metabolic reprogramming of CAFs constitutes an additional mechanism of chemotherapeutic resistance. The “reverse Warburg effect” describes a metabolic coupling in which CAFs undergo aerobic glycolysis, generating lactate, pyruvate, and ketone bodies that are shuttled to adjacent cancer cells to fuel oxidative phosphorylation and anabolic biosynthesis [56,57]. In gastric cancer, Kogure et al. showed that cancer cells with high metastatic potential induced a glycolytic shift in co-cultured fibroblasts, promoting a shift from oxidative phosphorylation to aerobic glycolysis, with elevated lactate production and oxygen consumption, consistent with the reverse Warburg model [58,59]. Bonuccelli et al. provided complementary evidence that ketones and lactate can fuel tumor growth and metastasis through this metabolic symbiosis [60].

CAF-derived exosomes further contribute to metabolic resistance through glutamine transfer. Sazeides and Le described a mechanism whereby CAF-derived exosomes augment glutamine consumption in cancer cells; ammonia, a by-product of glutamine metabolism, stimulates autophagy in CAFs, generating additional glutamine in a positive-feedback loop that sustains cancer cell growth and chemoresistance [61].

Amino acid depletion within the TME also impairs T cell function and drug efficacy. Tryptophan catabolism through the Indoleamine 2,3-dioxygenase 1 (IDO1) and tryptophan 2,3-dioxygenase 2 (TDO2)-kynurenine pathways suppresses anti-tumor immunity and promotes cancer progression, representing a metabolic vulnerability that intersects with both immune evasion and therapeutic resistance [62].

### 3.3. Exosome and Extracellular Vesicle-Mediated Resistance

CAF-derived EVs transfer bioactive cargo that directly promotes chemoresistance. In esophageal cancer, multiple studies have demonstrated that exosomal miR-21 reduces cisplatin sensitivity. Wan et al. showed that human esophageal fibroblast-derived exosomal miR-21 decreased cisplatin sensitivity in EC9706 cells by downregulating PTEN and PDCD4 [63]. Zhao et al. further demonstrated that CAFs promote cisplatin resistance in esophageal SCC through a dual mechanism involving both IL-6 and exosome-packaged miR-21, which synergistically activate STAT3 signaling to induce monocytic myeloid-derived suppressor cell generation [64]. While CAF-secreted miR-146a has been less extensively studied in upper GI cancers, Zhuang et al. demonstrated that CAF-derived miR-146a-5p promotes stemness and chemoresistance to gemcitabine and cisplatin in bladder cancer, suggesting a potentially conserved mechanism across tumor types [65].

Exosomal packaging of structural proteins also mediates resistance. Uchihara et al. demonstrated that CAF-derived EVs containing Annexin A6 induced drug resistance in gastric cancer cells by stabilizing β1 integrin at the cell surface, thereby activating focal adhesion kinase (FAK) and downstream YAP signaling. This promoted tubular network formation in the ECM, and inhibition of FAK or YAP efficiently attenuated gastric cancer drug resistance both in vitro and in a peritoneal metastasis mouse model [66]. Complementary work by Leca et al. in PDAC identified an ANXA6/LRP1/TSP1 complex in CAF-derived EVs that enhanced tumor cell survival and migration, with ANXA6 depletion in CAFs impairing tumorigenesis and metastasis [67].

### 3.4. Immune Exclusion

Immune exclusion represents a critical mechanism by which CAFs sustain tumor proliferation despite immunotherapy. The CXCL12/CXCR4 axis, driven predominantly by iCAFs, creates immunosuppressive gradients within the TME. Qin et al. demonstrated that high CXCL12 expression in CAFs significantly correlated with histological grade, TNM stage, and poor overall survival in gastric cancer patients. CAF-derived CXCL12 promoted migration, invasion, and EMT of gastric cancer cells, and blocking CXCL12 in CAFs diminished downstream Wnt5a and β-catenin expression [68]. Izumi et al. further showed that CXCL12/CXCR4 activation by CAFs mediated integrin β1 clustering at the cell surface, promoting invasive capacity that was efficiently inhibited by the CXCR4 antagonist AMD3100 [69].

Direct exclusion of cytotoxic T cells from tumor nests further compromises anti-tumor immunity. Wang et al. demonstrated that PDE5A-positive CAFs produce a TME that suppresses immune responses by promoting T cell exclusion and reducing CD8+ cytotoxic T cell infiltration into malignant tissue, contributing to immunotherapy resistance in gastric cancer [70].

PD-L1 upregulation on CAFs constitutes an additional checkpoint resistance mechanism. Kawasaki et al. demonstrated in esophageal cancer that PD-L1-expressing CAFs were associated with worse survival outcomes. Direct interaction between cancer cells and CAFs mutually upregulated PD-L1 expression, and anti-PD-L1 antibody treatment increased CD8+ T cell infiltration, decreased regulatory T cells, and induced death of both cancer cells and CAFs in vivo [71]. Wei et al. further elucidated that fibroblast activation protein (FAP) impedes degradation of STAT1 protein in CAFs, thereby sustaining PD-L1 transcription and fostering T cell exhaustion, with PD-L1 neutralizing antibodies effectively attenuating FAP-mediated immunosuppression [72].

### 3.5. Direct Contact-Mediated Resistance

Physical interactions between CAFs and cancer cells provide an additional layer of therapeutic resistance. Tang et al. demonstrated that connexin 43 (Cx43) mediates heterocellular gap junctional intercellular communication between gastric cancer cells and peritoneal mesothelial cells. Cx43-expressing gastric cancer cells formed functional heterocellular gap junctions with mesothelial monolayers within one hour, and a significant increase in diapedesis was observed compared with controls, occurring via a paracellular route [73]. This mechanism facilitates peritoneal metastasis and immune evasion.

N-cadherin-mediated survival signaling provides additional contact-dependent resistance. Ni et al. identified cadherin 11 as a mediator of fibroblast binding to gastric cancer cells, activating malignant potential through Yes-associated protein (YAP)-tenascin C (TNC) signaling and promoting metastasis [74]. In non-small cell lung cancer, Yamauchi et al. demonstrated that N-cadherin expression confers a survival advantage against gefitinib (an EGFR TKI) through PI3K/Akt signaling, suggesting a conserved mechanism by which cadherin-mediated cell–cell contacts promote resistance to targeted therapies across cancer types [75].

## 4. ECM Remodeling as a Pharmacological Barrier in Upper GI Cancers

### 4.1. Composition of the Desmoplastic Stroma in Upper GI Cancers

Desmoplasia, defined by the extensive overproduction of ECM components, is a hallmark of upper GI cancers [22]. The desmoplastic stroma results from complex and dynamic ECM remodeling, and its formation follows a stereotyped progression analogous to wound repair. Initially, tumor oxygen demands drive rapid and disorganized angiogenesis, leading to the deposition of a fibrin-rich scaffold constituting the early provisional matrix. Fibrin promotes an inflammatory milieu by activating macrophages and upregulating pro-inflammatory cytokines such as IL-1β, tumor necrosis factor (TNF)-α, and IL-6 [76]. Fibronectin is then deposited in the interstitial space and cross-linked to the fibrin network. Subsequently, invading fibroblasts secrete and assemble an elaborate fibronectin-rich transitional ECM (the late provisional matrix), which serves as a growth factor depot and mechanotransductive scaffold [77].

The extent of desmoplasia differs considerably across upper GI cancers. PDAC is the most extreme example, where non-malignant stroma, dominated by activated PSCs, can comprise majority of the total tumor volume [78,79]. Gastric cancer displays greater variability, with stromal content ranging from moderate to extensive depending on histological subtype; diffuse-type gastric cancers harbor significantly denser CAF infiltration and higher stromal gene expression profiles than intestinal-type tumors, with stroma-rich tumors carrying a markedly worse prognosis independent of subtype [80,81]. On the other hand, esophageal adenocarcinoma is characterized by a predominantly myofibroblastic, CAF-rich stroma present in the vast majority of tumors, while esophageal SCC also displays high stromal infiltration, with stroma-high tumors associated with reduced survival in both subtypes [82,83]. These differences in stromal burden have direct implications for drug penetration and the relative contribution of ECM remodeling to therapeutic resistance across these three tumor types.

In parallel, matricellular proteins such as periostin (POSTN) stabilize this scaffold by bridging collagen I, fibronectin, and tenascin-C. In esophageal SCC, POSTN derived from CAFs promotes tumor cell survival, migration, and invasion through integrin-mediated activation of PI3K-Akt and ERK signaling pathways [84,85]. In gastric cancer, CAF-derived POSTN promotes tumor growth through ERK activation and is clinically associated with greater invasion depth, lymph node metastasis, advanced stage, and poor overall survival [86,87].

Collagen expression is significantly upregulated in pancreatic and gastric cancers [88]. Two families of enzymes orchestrate collagen modification in upper GI tumorigenesis. First, lysyl hydroxylase 2 (encoded by the *PLOD2* gene), which mediates the formation of stable collagen cross-links, is upregulated in esophageal SCC and gastric cancer and is associated with poorer prognosis [89,90]. In esophageal SCC, PLOD2 expression correlates with T classification, lymph node metastasis, and pathological stage, and combined PLOD2 and ZEB1 expression serves as an independent prognostic factor [89]. In gastric cancer, high PLOD2 expression is significantly associated with peritoneal dissemination and poor survival, regulated by hypoxia-inducible factor-1 (HIF-1) [91]. Second, lysyl oxidase (LOX) and lysyl oxidase-like 2 (LOXL2), which deaminate lysine residues in tropocollagen to initiate cross-linking, stabilize collagen into dense fibrils [92]. LOXL2 is overexpressed in gastric carcinoma, where stromal LOXL2 expression independently predicts overall survival and promotes cancer cell motility via the Src/FAK pathway [93,94]. In esophageal SCC, LOXL2 promotes cytoskeletal reorganization and tumor cell invasion through phosphorylation of ezrin, and high expression correlates with poor clinical outcome [95].

Finally, hyaluronic acid (HA), a glycosaminoglycan synthesized by CAFs and tumor cells, generates a viscous gel phase that swells the matrix [96,97]. The mature desmoplastic stroma is ultimately characterized by a dense ECM composed primarily of collagen, fibronectin, proteoglycans, and HA, which produces a mechanically hostile niche.

### 4.2. Biophysical Drug Exclusion

The biophysical properties of the desmoplastic stroma limit tumor drug delivery through two principal mechanisms: elevated interstitial fluid pressure (IFP) and collagen-mediated diffusion restriction [98]. In PDAC, IFP values up to 100 mmHg have been detected—far exceeding arteriolar pressure of 40–80 mmHg—thereby collapsing tumor vasculature and abolishing the transcapillary inward pressure gradient required for drug delivery [99,100]. Preclinical studies have identified HA as the key ECM component responsible for this elevated IFP through the creation of a gel-fluid phase; enzymatic degradation of HA with PEGylated recombinant hyaluronidase (PEGPH20) significantly reduces interstitial pressure, re-expands collapsed vasculature, and improves delivery of concurrently administered agents [99,100,101]. Spatial studies in PDAC have demonstrated that tissue pressure is maximized in areas where HA expansion is restricted by dense collagen networks, creating high-stress microregions [102].

As a result, diffusion becomes the sole mechanism for systemic drug delivery. However, densely packed collagen fibers form a diffusion barrier, particularly for larger molecules, further hindering drug penetration [76,103]. Collagen content is substantially elevated in PDAC and gastric carcinoma compared with their normal tissue counterparts [76]. Furthermore, polarization-resolved second harmonic generation (SHG) microscopy has revealed higher disorder of collagen ultrastructure within gastric carcinoma and PDAC, reflecting the pathological remodeling that characterizes these tumors [104,105].

### 4.3. ECM Stiffness and Mechanotransduction Driving Resistance

Beyond impeding drug delivery, desmoplastic stroma stiffness also drives tumor drug resistance through mechanosensory signaling cascades. PDAC is among the stiffest solid tumors owing to its dense desmoplastic stroma, though reported stiffness values depend strongly on measurement method and scale. By atomic force microscopy, PDAC tissue ranges from roughly 0.5–15 kPa (with most values between 1 and 10 kPa) versus approximately 0.5 kPa for normal pancreas [106]. Viscoelastic rheometry similarly reports mean tumor moduli of 5.46 ± 3.18 kPa, compared with 1.06 ± 0.25 kPa in normal tissue and 2.15 ± 0.41 kPa in pancreatitis [107]. This rigidity is sensed through integrins at focal adhesions, activating the FAK-PI3K-Akt-mTOR axis and promoting pro-survival signaling. Specifically, type I collagen activates integrin β1 (ITGB1) in gastric cancer to upregulate anti-apoptotic signaling, while type V collagen-ITGB1 interaction promotes PDAC proliferation and invasiveness [108]. Furthermore, ITGB1 plays an essential role in anoikis resistance by activating the PI3K-Akt pathway, providing an E-cadherin-independent survival mechanism during invasion [109,110]. ITGB1 promotes resistance to gemcitabine in PDAC through multiple mechanisms, including inhibition of ferroptosis via the FAK/AKT/mTOR-GPX4 cascade, reduced drug uptake through integrin α3β1-mediated suppression of the gemcitabine transporter ENT1, and activation of the PI3K-Akt axis to promote cell survival during chemotherapy [111]. Similarly, ITGB1 has been shown to mediate resistance to cisplatin and docetaxel in gastric carcinoma and esophageal SCC [110].

The downstream transcriptional co-activators YAP and TAZ act as the principal effectors of mechanosensing in tumor cells. Dupont et al. demonstrated that matrix rigidity alone is sufficient for YAP/TAZ nuclear translocation, independently of soluble growth factors [112]. In normal tissues, the Hippo pathway acts as a tumor suppressor and inactivates YAP/TAZ. However, ECM stiffness disrupts this pathway and promotes YAP/TAZ nuclear translocation [113,114]. YAP/TAZ function as oncoproteins driving cancer progression through tumor proliferation, invasion, EMT, and stemness properties, and they also drive resistance to chemotherapy and radiation in upper GI cancers [114]. It is worth mentioning that in PDAC, Yang et al. demonstrated that YAP1-driven tumor-like CAFs (tCAFs) promote ECM stiffness and EMT, and combination of the YAP1 inhibitor verteporfin with gemcitabine suppressed tCAF function, reduced stromal stiffness, and improved survival in KPC mouse models [115].

Importantly, YAP/TAZ activation confers mechanical memory to cancer cells, maintaining resistance mechanisms even after resolution of ECM rigidity. Under high-stiffness conditions, METTL14 enhances YAP1 expression through YTHDF3-mediated m6A-dependent translational regulation, while YAP1 in turn transcriptionally upregulates METTL14 via TEAD1, establishing a positive feedback loop that sustains mechanical memory and drives stromal remodeling through the CD166-EGFR-LOXL2 signaling axis [116]. Similarly, ECM rigidity leads to epigenetic regulation of YAP expression, contributing to sustained oncogenic activation in gastric cancer [117].

### 4.4. Matrix Metalloproteinases: A Double-Edged Sword

Matrix metalloproteinases (MMPs) play a paradoxical role in upper GI cancers. These zinc-dependent endopeptidases are capable of degrading all ECM components, facilitating tumor invasion through the stiff stroma and metastasis. MMP-2 drives proteolysis of type IV collagen and basement membrane constituents, while MMP-7 cleaves β4 integrin and E-cadherin to further erode adhesion barriers, easing tumor escape [118,119]. In gastric cancer, MMP-2 and MMP-7 expression correlates significantly with depth of invasion, lymph node metastasis, and poor prognosis [120,121].

However, MMP-mediated ECM proteolysis introduces a breach into the high-pressure, diffusion-restricted desmoplastic stroma. MMPs can transiently lower IFP in peri-tumoral tissue and reduce diffusion barriers, potentially improving drug delivery [118,122]. Hence, targeting these enzymes poses a therapeutic challenge, requiring a careful balance between inhibiting tumor metastasis and preserving drug delivery pathways. The failure of broad-spectrum MMP inhibitors in clinical trials (due to severe dose-limiting side effects such as musculoskeletal syndrome) underscores this complexity and highlights the need for more selective therapeutic strategies [123].

## 5. Spatial Multi-Omics: Mapping Drug Resistance Architecture in the Upper GI Tumor Microenvironment (TME)

The use of spatial profiling allows for additional information to be introduced for analysis when tissues are dissolved, homogenized, or prepared for bulk analysis or single-cell sequencing. The reason for this is that this technique allows for better retention of the positions of molecularly defined cells, allowing for better association with tissue architecture which, in turn, allows for a more detailed analysis of spatially organized CAF populations and tumor-stroma interfaces.

### 5.1. Spatial Profiling Technologies

Several complementary platforms now enable spatially resolved molecular profiling of the TME (Table 2). Sequencing-based approaches such as 10x Genomics Visium provide whole-transcriptome spatial mapping at a resolution of 55 µm spots, each capturing transcripts from approximately 1–10 cells [124]. While this provides broad transcriptomic coverage across tissue regions, it averages the molecular signatures of heterogeneous cell populations within each spot, potentially obscuring distinctions between cancer cells, fibroblasts, and immune cells.

The benefit of sequence-based spatial transcriptomics lies in the ability to allow for broad transcriptome-level discoveries across different tissue regions; however, while these benefits are evident, this technique is limited by the presence of more than one cell type in spot measurements. This therefore requires further steps including histological techniques to further estimate the composition of samples [124,125,126,127].

Imaging-based spatial transcriptomics platforms, including MERFISH (Vizgen MERSCOPE), CosMx (NanoString), and Xenium (10x Genomics), achieve true single-cell or subcellular resolution by detecting hundreds to thousands of RNA transcripts simultaneously within intact tissue architecture [128]. Systematic benchmarking across cancer types has demonstrated that these platforms differ in transcript capture sensitivity, cell segmentation accuracy, and spatial signal fidelity, with Xenium consistently generating higher transcript counts per gene and CosMx and Xenium showing the strongest concordance with orthogonal single-cell transcriptomics [129,130]. These targeted platforms enable precise localization of drug-resistant clones and their stromal neighbors within the tissue context. The shortcoming that exists with these platforms, however, is that they give up the broad and less biased coverage that sequencing-based approaches use because they instead measure at more predefined probe levels. Despite this shortcoming, they use said probe levels to allow for precise subcellular localization [128,129,130].

Spatial proteomics platforms complement transcriptomic approaches by enabling simultaneous visualization of dozens of protein markers. Co-detection by indexing (CODEX, now Akoya PhenoCycler) uses cyclical immunofluorescence with oligonucleotide-barcoded antibodies to detect over 100 proteins at subcellular resolution [131]. Multiplexed Ion Beam Imaging (MIBI) employs metal isotope-tagged antibodies and time-of-flight mass spectrometry to simultaneously quantify more than 36 proteins at subcellular resolution [132,133]. These proteomic tools are particularly suited for analyzing the functional states of CAFs and the spatial distribution of structural ECM proteins that constitute physical barriers to drug delivery and immune cell infiltration. CODEX and MIBI are both platforms that can preserve tissue organization while simultaneously allowing for multiplex protein phenotyping as well as cell neighborhood analysis. These platforms remain limited by the specificity and quality of antibodies selected. Additionally, the molecular coverage of these platforms is also limited by overall panel design [131,132,133].

Despite their considerable power, imaging-based spatial transcriptomics and proteomics platforms carry important technical limitations that are particularly relevant in the desmoplastic context of upper GI tumors. Because these methods rely on fluorescence signal detection within intact tissue sections, highly desmoplastic or necrotic regions pose a major challenge: FFPE tissues contain abundant auto-fluorescent molecules, including fibrotic collagen, blood cells, and other endogenous chromophores, that generate broad-spectrum background emission spanning 400–600 nm, reducing signal-to-noise ratios and compromising probe detection accuracy [134]. In addition, RNA degradation in archival FFPE samples further affects transcript recovery, with amplification-dependent platforms showing greater robustness to degraded material than unamplified approaches [130].

### 5.2. Gastric Cancer: myCAF Niches and Drug-Resistant Clones

In gastric cancer, spatial multi-omics has delineated a more heterogeneous conception of the tumor stroma, revealing organized niches in which distinct CAF subpopulations actively govern tumor survival. Wang et al. integrated 14 independent scRNA-seq datasets comprising 239 GI adenocarcinoma samples and applied spatial transcriptomics to demonstrate that two myofibroblastic CAF subtypes (myCAF1 and myCAF2) preferentially occupied discrete spatial domains within the TME [135]. These myCAF-driven stromal niches were consistently associated with poor prognosis, characterized by ECM remodeling, TGF-β signaling, hypoxia adaptation, and close crosstalk with immunosuppressive macrophages and tumor cells displaying EMT signatures [135]. Spatial profiling confirmed that myCAFs, characterized by high expression of α-SMA and FAP, form densely structured niches enveloping tumor nests rather than randomly permeating the tumor bed [136,137].

Within these niches, myCAFs secrete dense ECM components that physically protect gastric cancer cells from cytotoxic agents, while paracrine survival signals including TGF-β and CXCL12 further sustain drug-resistant clones [45,138]. Li et al. demonstrated through single-cell sequencing of gastric cancer peritoneal metastases that a stroma-myeloid niche composed of Thrombospondin 2 (THBS2)+ matrix CAFs and SPP1+ tumor-associated macrophages was the major mediator of immune checkpoint blockade resistance, and that blocking the C3-C3AR1 axis disrupted this niche and significantly improved immunotherapy benefit in vivo [139]. These findings establish that the spatial co-localization of specific CAF subtypes with tumor cells creates microenvironments of high interstitial pressure and hypoxia that physically exclude chemotherapeutic agents and enable drug-resistant clones to thrive.

### 5.3. Esophageal Cancer: Barrett’s-to-Adenocarcinoma ECM Evolution

The transition from Barrett’s esophagus to esophageal adenocarcinoma (EAC) represents a well-characterized architectural transformation driven by progressive ECM evolution. Strasser et al. performed multi-omic profiling integrating single-cell transcriptomics, ECM proteomics, tissue mechanics, and spatial proteomics across the progression from squamous epithelium through metaplasia, dysplasia, and adenocarcinoma in 107 samples from 26 patients [140]. This analysis revealed that metaplastic replacement of epithelial cell composition was paralleled by coordinated changes in stromal cells, ECM composition, and tissue stiffness. Fibroblasts with the molecular characteristics of CAFs were already present in precancerous metaplasia, producing POSTN, whose expression shifted from vascular to stromal cells, consistent with the emergence of an immunosuppressive microenvironment enriched for regulatory NK and Treg cells [140].

Cruz-Acuña et al. developed an engineered tumor ECM-mimetic hydrogel platform with tunable mechanical properties and demonstrated that matrix stiffness controls EAC organoid formation, growth, proliferation, and activation of tumor-associated pathways that elicit stem-like properties, both in vitro and in vivo [141]. Complementary gene expression analyses have revealed significant enrichment of ECM matrisome gene sets in dysplastic Barrett’s esophagus and EAC compared with controls, with progressive loss of basement membrane markers such as agrin (AGRN) during neoplastic progression [142]. This shows that the malignant transition involves a progressive spatial realignment from an immunopermissive matrix to a dense, immunosuppressive barrier that impedes both neoadjuvant chemoradiotherapy and immune checkpoint inhibitors.

### 5.4. Pancreatic Cancer: Stellate Cell Core and Immune Rim Architecture

PDAC serves as the archetype for stroma-driven resistance, with non-malignant stromal elements comprising the volumetric majority of tumor tissue [143]. Spatial multi-omics has revealed a profound compartmentalization of the PDAC TME, with a desmoplastic core dominated by activated pancreatic stellate cells (PSCs) that effectively wall off the tumor from immune surveillance [144,145].

Grünwald et al. deconvoluted the human pancreatic TME through large-scale integration of histology-guided regional multi-omics with clinical data and discovered “subTMEs”—histologically definable tissue states anchored in fibroblast plasticity. Matrix-rich “deserted” subTMEs harbored fewer activated fibroblasts yet were markedly chemoprotective and enriched upon chemotherapy, while “reactive” subTMEs rich in complex fibroblast communities were immune-hot but inhabited by aggressive tumor cell phenotypes [146]. Du et al. further demonstrated that PLXDC1+ tumor-associated PSCs, exhibiting a myCAF phenotype, formed a desmoplastic and immunosuppressive niche around the tumor boundary in close proximity to aggressive LRRC15+ myCAFs and SPP1+ macrophages, promoting CD8+ T cell exhaustion and correlating with poor immunotherapy efficacy [144].

This spatial exclusion exemplifies why systemic therapies frequently fail, where the drug-resistant architecture physically segregates therapeutic agents and immune effectors from malignant epithelial cells [145,147]. Effector T cells are largely confined to the tumor periphery, trapped in dense collagenous tracts and unable to penetrate the PSC-dominated core.

### 5.5. Integrating Spatial and Single-Cell Data to Map Resistance Niches

Computational deconvolution methods increasingly enable the integration of spatial transcriptomics with dissociated scRNA-seq data to identify resistance niches at high resolution [148]. Tools such as SPOTlight, STRIDE, Cell2location, and Redeconve map deep, unbiased scRNA-seq phenotypic data onto the geographic coordinates provided by spatial platforms, enabling precise identification of cell-type composition within spatially defined microenvironments [125,126,127]. Li et al. benchmarked 18 deconvolution methods across 50 datasets and identified CARD, Cell2location, and Tangram as the most accurate approaches for this task [125].

By anchoring scRNA-seq data spatially, it becomes possible not only to identify which resistance pathways are activated in specific tumor and stromal subclones, but to determine exactly where these cells reside relative to vasculature, immune infiltrates, and dense ECM tracts [148,149]. Application of Redeconve to human pancreatic cancer datasets has revealed cancer-clone-specific T cell infiltration patterns, providing novel insights into the spatial determinants of immune evasion [150].

### 5.6. Translational Potential: Spatial Biomarkers for Treatment Prediction

The spatial architectures identified by multi-omics platforms hold considerable promise as predictive biomarkers (Table 3). Rather than relying solely on bulk mutational burden or isolated protein expression, emerging approaches quantify spatial scores, such as the proximity of myCAFs to tumor islands, the density of the immune-excluded ECM boundary, or the composition of stromal-immune niches, to predict treatment response [151,152]. Furthermore, potential clinical uses involve the stratification of patients by spatial stromal or immune phenotypes and then the development of tissue-based mechanisms of predicting therapeutic response. These are potential applications that still stand to be investigated and validated [151,152,153].

Xu et al. performed digital spatial profiling on pre-treatment biopsies from patients with locally advanced gastric cancer treated with immunochemotherapy and identified spatially resolved biomarkers, including NOTUM, SERPINA3, and CD8+ T cell density within specific tissue compartments, that strongly predicted a major pathological response [151]. Kim et al. demonstrated in resected PDAC that AI-powered spatial analysis of immune phenotypes (classifying tumors as immune-inflamed, immune-excluded, or immune-desert) provided independent prognostic information beyond conventional pathological staging, with the immune-excluded phenotype predominating in most patients [153].

These advances suggest that identifying spatial configurations in pre-treatment biopsies may ultimately enable clinicians to predict treatment failure before it occurs and to rationally select combination strategies that remodel the TME to overcome resistance. For example, the same biopsy material used for spatial multi-omics profiling can be used to generate three-dimensional Patient-derived organoids (PDOs) that preserve the phenotypic and genotypic characteristics of the original tumor, enabling functional drug-response testing in real time. Vlachogiannis et al. established a living biobank of PDOs from metastatic, heavily pre-treated colorectal and gastroesophageal cancer patients enrolled in phase 1/2 clinical trials and demonstrated that ex vivo drug-response profiles in PDOs correlated with patient clinical outcomes, establishing PDOs as a tractable platform for predicting treatment response in upper GI malignancies [154].

## 6. Therapeutic Strategies Targeting CAF Heterogeneity and ECM Remodeling

### 6.1. Anti-CAF Approaches

FAP, a type II transmembrane serine protease overexpressed on activated CAFs, has emerged as a leading therapeutic target. FAP modulates the TME by promoting tumor angiogenesis, immunosuppression, and ECM remodeling. Several FAP-directed strategies are under active investigation. FAP-targeting chimeric antigen receptor (CAR) T cells have demonstrated preclinical efficacy in PDAC, where in vivo FAP-CAR macrophages reduced activated CAF markers, collagen volume fraction, and type I collagen secretion by 3- to 5-fold in orthotopic PDAC models, enhancing penetration of gemcitabine and immune cells and significantly prolonging survival [155]. Dual-targeting FAP/CLDN18.2 CAR-T cells suppressed myeloid-derived suppressor cell recruitment and reduced T cell exhaustion in a TGF-β-dependent manner in PDAC models [156,157]. FAP-targeting antibody-drug conjugates (ADCs) have also shown promise. For instance, OMTX705, a FAP-targeting ADC, demonstrated activity in chemotherapy- and pembrolizumab-resistant solid tumor models, including pancreatic cancer. A clinical trial evaluating OMTX705 with or without anti-PD-1 therapy is currently ongoing (NCT05547321) [158]. While these approaches remain largely in preclinical and early clinical phases, they highlight the therapeutic potential of depleting or modulating FAP-expressing CAFs to overcome stromal barriers.

iCAF targeting via IL-6 receptor/JAK inhibition represents another approach. iCAFs support tumor growth, immune suppression, and treatment resistance through the secretion of IL-6, CXCL1, and other inflammatory mediators. However, clinical translation has been challenging, and response to iCAF-targeted strategies in PDAC has been modest, warranting further investigation of optimal patient selection and combination approaches.

Hedgehog pathway inhibition, initially pursued to deplete the desmoplastic stroma produced by myCAFs, has yielded cautionary results. Smoothened inhibitors such as vismodegib and IPI-926 (saridegib) showed preclinical promise by reducing tumor stroma and improving chemotherapy delivery [159]. However, clinical trials failed to demonstrate benefit. A randomized phase Ib/II trial of vismodegib plus gemcitabine showed no improvement in progression-free survival (PFS), overall survival (OS), or response rate compared with gemcitabine plus placebo (median PFS 4.0 vs. 2.5 months; median OS 6.9 vs. 6.1 months) [160]. A separate phase II trial of vismodegib combined with gemcitabine and nab-paclitaxel similarly showed no improvement over historical chemotherapy-alone rates [159].

Preclinical studies by Rhim et al. revealed that hedgehog pathway inhibition, while reducing stroma, produced tumors with increased vascularization, more undifferentiated histology, and higher proliferative indices, indicating that certain stromal elements may restrain rather than promote tumor progression. [161]. Steele et al. further demonstrated that hedgehog inhibition shifts CAF composition from myCAFs to iCAFs, correlating with decreased cytotoxic T cells and expanded regulatory T cells, consistent with increased immunosuppression. [162]. These findings fundamentally reshaped the understanding of tumor-stroma interactions and underscore that indiscriminate stromal depletion may be counterproductive.

### 6.2. ECM Normalization Strategies

Given the risks of complete stromal ablation, ECM normalization (“softening” the matrix to improve therapeutic penetration without eliminating its tumor-restraining functions) has emerged as an alternative concept. PEGPH20 (pegvorhyaluronidase alfa), a PEGylated recombinant human hyaluronidase, was developed to degrade HA and reduce interstitial fluid pressure. A randomized phase II trial (HALO 202) showed improved PFS with PEGPH20 plus nab-paclitaxel/gemcitabine, particularly in patients with HA-high tumors (HR 0.51; 95% CI, 0.26–1.00; *p* = 0.048) [163]. However, the definitive phase III HALO 109–301 trial in 494 patients with HA-high metastatic PDAC demonstrated no improvement in OS (11.2 vs. 11.5 months; HR 1.00; 95% CI, 0.80–1.27; *p* = 0.97) or PFS (7.1 vs. 7.1 months), despite a higher objective response rate (47% vs. 36%) [164]. A separate phase Ib/II trial (SWOG S1313) of PEGPH20 combined with modified FOLFIRINOX was closed early for futility, with a median OS of 7.7 months in the combination arm versus 14.4 months with FOLFIRINOX alone, suggesting potential detriment [165]. These results led to the suspension of further development and exploration of PEGPH20 in PDAC [166].

Simtuzumab, a humanized monoclonal antibody targeting LOXL2, was investigated to inhibit collagen cross-linking and reduce matrix stiffness. A phase II randomized, double-blind, placebo-controlled trial of 240 patients with metastatic PDAC showed no improvement in PFS (3.7 vs. 3.5 vs. 3.7 months for simtuzumab 700 mg, 200 mg, and placebo, respectively) or OS with the addition of simtuzumab to gemcitabine [167]. Subsequent mechanistic studies revealed that simtuzumab (the humanized version of AB0023) does not actually inhibit LOXL2 catalytic activity or collagen cross-linking but rather exerts anti-angiogenic effects through a non-enzymatic mechanism, potentially explaining the clinical failure [168].

Inhibition of the TGF-β pathway has shown more encouraging signals. Galunisertib, a first-in-class oral TGF-β receptor I (ALK5) kinase inhibitor, was evaluated in combination with gemcitabine in a randomized phase II trial of 156 patients with unresectable pancreatic cancer. The combination improved median OS compared with placebo plus gemcitabine (8.9 vs. 7.1 months; HR 0.79), with minimal added toxicity [169]. A next-generation ALK5 inhibitor, vactosertib, is currently being evaluated in combination with standard therapy in an active clinical trial (NCT06044311) [170].

### 6.3. Mechanotransduction Targeting

Collagen and fibrotic ECM deposition activate mechanosensing pathways, principally focal adhesion kinase (FAK) and YAP/TAZ signaling, that promote further fibrosis, tumor development, and stromal reinforcement. Jiang et al. demonstrated in the KPC mouse model (p48-Cre;LSL-Kras(G12D);Trp53(flox/+); genetically engineered mouse model designed to study PDAC) that focal adhesion kinase (FAK) inhibition reduced tumor fibrosis, decreased immunosuppressive cell infiltration, and rendered PDAC responsive to PD-1 checkpoint immunotherapy [171]. Clinically, Wang-Gillam et al. conducted a phase I dose-escalation and expansion study of defactinib (a selective oral FAK inhibitor) combined with pembrolizumab and gemcitabine in patients with advanced treatment-refractory pancreatic cancer. The triple combination was well tolerated with no dose-limiting toxicities. Among 20 patients with refractory PDAC, the disease control rate was 80%, with a median PFS of 3.6 months and median OS of 7.8 months. Pre- and post-treatment biopsies demonstrated increased infiltrative T lymphocytes [172,172]. A randomized phase II neoadjuvant trial further showed that pembrolizumab combined with defactinib was associated with lower FAP+ fibroblast infiltration, a 5.44-fold increase in CD8+ T cell percentage (vs. 2.01-fold with pembrolizumab alone; *p* = 0.02), and significant increases in anti-tumor M1 macrophages [173]. The combination of avutometinib (a RAF/MEK clamp) with defactinib plus gemcitabine/nab-paclitaxel is being evaluated in the ongoing RAMP 205 phase Ib/2 trial (NCT05669482), with preliminary data showing a 75% partial response rate among efficacy-evaluable patients and 100% disease control rate [174,175].

Verteporfin, a YAP/TAZ-TEAD interaction inhibitor, has demonstrated preclinical anti-tumor activity in gastric cancer. Giraud et al. showed that verteporfin inhibited YAP/TAZ-TEAD transcriptional activity, reduced CD44+ cancer stem cell populations, and inhibited tumor growth in patient-derived gastric cancer xenograft models [176]. Hasegawa et al. confirmed that verteporfin inhibited gastric cancer cell proliferation in a dose-dependent manner by suppressing the anti-apoptotic protein survivin in both YAP-dominant and TAZ-dominant cell lines [177]. Nam et al. further demonstrated that verteporfin effectively reduced tumor growth and increased apoptosis in trastuzumab-resistant HER2-positive gastric cancer xenograft models, suggesting its potential to overcome targeted therapy resistance [178]. However, clinical translation of YAP/TAZ inhibitors remains in early stages.

### 6.4. Nanomedicine for Stroma-Penetrating Drug Delivery

The dense collagen barrier in PDAC impedes penetration of conventional chemotherapeutic agents, motivating the development of ECM-degrading nanomedicine approaches. Zinger et al. demonstrated that pretreatment with collagenase-encapsulated liposomes (“collagozomes”) reduced fibrotic tissue from 12.8% to 5.6% volume in PDAC-bearing mice, and subsequent treatment with paclitaxel micelles yielded tumors 87% smaller than controls, without increasing circulating tumor cells or metastasis [179]. Hwang et al. recently reported gemcitabine-loaded collagozomes that achieved 6-fold higher tumor growth inhibition (69.8%) compared with non-functionalized liposomes (10.9%) in PDAC models, with the first molecular-level validation of nanocarrier-mediated drug penetration using desorption electrospray ionization mass spectrometry imaging [180]. Zhao et al. developed collagenase nanogel backpacks that bind CAR-T cells via CXCR4 antagonist peptides, modulating tumor infiltration by surmounting physical barriers and disrupting chemokine-mediated CAR-T cell imprisonment, thereby improving CAR-T cell therapy outcomes in pancreatic cancer [181].

Losartan, an angiotensin II receptor blocker with antifibrotic properties, has been investigated as a stromal decompression agent. Murphy et al. conducted a single-arm phase II trial of neoadjuvant FOLFIRINOX plus losartan, followed by chemoradiotherapy, in 49 patients with locally advanced pancreatic cancer, achieving a 61% R0 resection rate and a median OS of 31 months [182]. Boucher et al. demonstrated that the addition of losartan downregulated immunosuppression and pro-invasion genes, reduced regulatory T cells, and increased CD8+ T cell infiltration in resected specimens [183]. However, the randomized AFPAC study of losartan plus modified FOLFIRINOX versus modified FOLFIRINOX alone in 88 patients with advanced PDAC showed no early signal of efficacy in improving PFS (HR 0.76; 95% CI, 0.47–1.22; *p* = 0.392), and the trial will not proceed to full accrual [184]. A phase II randomized trial incorporating losartan with FOLFIRINOX, nivolumab, radiation, and surgery is ongoing (NCT03563248) [185,186].

### 6.5. Combination Strategies and Active Clinical Trials

The convergence of ECM normalization, mechanotransduction inhibition, and nanomedicine approaches with immunotherapy represents a promising frontier. The rationale is supported by preclinical evidence demonstrating that stromal remodeling can convert immunologically “cold” tumors into “hot” microenvironments amenable to checkpoint blockade. Table 4 summarizes some active clinical trials targeting the stroma in upper GI cancers.

While significant challenges remain—including complex stromal and tumor heterogeneity, the dual tumor-promoting and tumor-restraining roles of the stroma, and the need for predictive biomarkers to guide patient selection—the growing body of preclinical and early clinical evidence supports the continued development of rationally designed combination strategies that remodel the TME to resensitize upper GI cancers to systemic treatment.

## 7. Conclusions

The high lethality of upper GI cancers reflects failure that may not be confined only to the cancer cells. Across esophageal, gastric, and pancreatic tumors, resistance to platinum chemotherapy, HER2- and VEGFR-directed agents, and immune checkpoint blockade emerges from a TME in which CAFs and the desmoplastic ECM act as active determinants of therapeutic outcome rather than inert scaffolding. The functionally distinct CAF programs that populate these tumors, together with the stiff, collagen-rich matrix they assemble, converge on a limited set of resistance mechanisms (paracrine survival signaling, metabolic coupling, extracellular vesicle transfer, immune exclusion, and biomechanical remodeling) that recur across all three anatomical sites despite their divergent epithelial origins. What unifies these cancers is therefore less a shared mutational landscape than a shared stromal logic, in which spatial organization, not molecular catalogue alone, dictates which therapies penetrate, which immune effectors engage, and which clones endure.

Recognizing this has reframed drug resistance as a property of tissue architecture. Spatial multi-omics now resolves the niches in which myofibroblastic fibroblasts sheath tumor nests, immune-excluded boundaries seal malignant cores, and stromal–myeloid compartments enforce checkpoint refractoriness. The repeated clinical failure of indiscriminate stromal ablation, set against early signals from approaches that instead reprogram fibroblast state or normalize matrix mechanics, indicates that the goal is not to remove the stroma but to recondition it. Realizing that goal will demand spatially resolved biomarkers capable of matching each patient to the resistance configuration their treatment is designed to dismantle, as well as combination strategies that pair microenvironmental remodeling with cytotoxic and immunological agents. The central challenge ahead is to translate a now-detailed map of where resistance resides into the means to dismantle it, and, in doing so, to convert these refractory cancers into ones that yield to the therapies we already possess.

## Figures and Tables

**Table 1:** Principal cancer-associated fibroblast (CAF) subtypes in upper gastrointestinal (GI) cancers, their markers, inducers, spatial localization, and roles in drug resistance.

CAF Subtype	Representative Markers	Key Inducers/ Signaling	Spatial Localization	Principal Role in Drug Resistance	Cancer-Type Specificity
myCAF (myofibroblastic)	α-SMA (ACTA2), TAGLN, THY1, IGFBP3, COL12A1, FAP	TGF-β	Tumor-proximal; envelops tumor nests	Desmoplastic ECM deposition and physical drug exclusion; TGF-β/CXCL12 paracrine survival signaling	PDAC: dominant population, largely PSC-derived, forms near-complete peritumoral sheath. Gastric: myCAF1/myCAF2 occupy discrete spatial domains. Esophageal: present already in Barrett‘s metaplasia, precedes overt malignancy.
iCAF (inflammatory)	IL-6, CXCL12, CXCL1, CCL7 (CXCL1/2/5/6, IL-11, MMP1/3/10 in gastric cancer)	IL-1α activate NF-κB and JAK-STAT3	Tumor-distal	Immunosuppression; IL-6/STAT3-mediated chemoresistance; CXCL12/CXCR4 immune exclusion	PDAC: site of the canonical IL-1α/TGF-β antagonism; the local cytokine ratio, not presence/absence, sets fate. Gastric: enriched in diffuse-type tumors with expanded chemokine/MMP repertoire. Esophageal SCC: induced via CXCL1-CXCR2-pSTAT3.
apCAF (antigen-presenting)	MHC class II (HLA-DRA, HLA-DRB1, CD74)	IL-1 and TGF-β (mesothelial MMT origin in PDAC)	Adjacent to cancer cells	Context-dependent: regulatory T-cell induction (PDAC) versus T-cell activation and improved ICI response (gastric cancer)	PDAC: mesothelial-derived, drives FoxP3+ Treg differentiation (net immunosuppressive). Gastric: net immunostimulatory, associated with improved ICI response—identical marker expression, opposite net effect.
mCAF (matrix)	POSTN (THBS2+ in peritoneal metastases)	Pericyte-derived progenitor	Matrix-rich stroma	Matrix remodeling; stroma–myeloid niche driving immune checkpoint blockade resistance	Gastric: THBS2+ mCAFs pair with SPP1+ TAMs in a stroma-myeloid ICB-resistance niche Not yet systematically resolved at spatial resolution in esophageal or pancreatic cohorts.
pCAF (proliferative)	TOP2A	Proliferative transcriptional program	Variable	Expansion of the activated CAF pool	Least characterized subtype across all three cancers; defined chiefly by a proliferation signature rather than a distinct secretory or matrix phenotype.

Abbreviations: ACTA2, actin alpha 2; CCL7, C-C motif chemokine ligand 7; CXCL, C-X-C motif chemokine ligand; FAP, fibroblast activation protein; HLA, human leukocyte antigen; ICI, immune checkpoint inhibitor; IL, interleukin; MHC, major histocompatibility complex; MMT, mesothelial-to-mesenchymal transition; PDAC, pancreatic ductal adenocarcinoma; POSTN, periostin; TGF-β, transforming growth factor β; THBS2, thrombospondin 2; TOP2A, DNA topoisomerase II alpha; α-SMA, α-smooth muscle actin.

**Table 2:** Characteristics and applications of spatial multi-omics platforms for profiling upper gastrointestinal (GI) cancers.

Platform	Spatial Resolution	Analyte/Capability	FFPE Compatibility	Advantages	Limitations	Typical CAF/ECM Applications	References
Visium (10x Genomics)	1–10 cells/spot. Spots are 55 µm in diameter. Visium HD has 2 µm continuous resolution	Whole transcriptomics	Yes	Unbiased whole-transcriptome coverage. Works on archival FFPE. Lower cost per sample.	Multi-cell spot averaging obscures CAF/immune/tumor boundaries. Requires computational deconvolution for near-single-cell resolution.	Whole-tumor stromal architecture mapping Input for deconvolution-based niche mapping (CARD, Cell2location, Tangram)	https://kb.10xgenomics.com/s/; https://www.10xgenomics.com/platforms/visium accessed on 10 July 2026
Xenium (10x Genomics)	Subcellular (single-molecule, single-cell segmentation) XY precision 50 nm; Z precision 100 nm	Targeted transcriptomics	Yes, FFPE-optimized	Highest transcript counts per gene among benchmarked imaging platforms Strong concordance with orthogonal scRNA-seq	Pre-selected gene panel limits discovery scope Sensitive to auto-fluorescent background in desmoplastic/necrotic tissue. Higher cost.	Precise localization of drug-resistant clones relative to myCAF/iCAF niches. In situ validation of scRNA-seq-derived CAF signatures	https://kb.10xgenomics.com/s/; https://www.10xgenomics.com/platforms/visium accessed on 10 July 2026
CosMx (NanoString)	Subcellular resolution (120 nm)	Targeted and whole transcriptomics and protein panels	Yes	Strong concordance with scRNA-seq. Combined RNA/protein panels feasible	Lower per-gene sensitivity than Xenium in some benchmarks	Joint RNA/protein spatial mapping of CAF-subtype markers alongside ECM transcripts	https://brukerspatialbiology.com/products/cosmx-spatial-molecular-imager/ accessed on 10 July 2026
MERFISH (Vizgen MERSCOPE)	Subcellular resolution (100 nm)	Targeted transcriptomics	FFPE via a dedicated FFPE workflow available.	High multiplexing via combinatorial barcoding. High spatial fidelity. Large field of view per run (up to 3 cm^2^ for MERSCOPE ULTRA)	Imaging time scales with panel size FFPE signal quality still maturing relative to fresh-frozen, still dependent on tissue quality	Fine-grained mapping of CAF-subtype gradients across the tumor-stroma interface	https://vizgen.com/ accessed on 10 July 2026
CODEX/PhenoCycler (Akoya)	Subcellular resolution (0.5 µm)	Targeted proteomic	Yes	Very high-plex protein detection.	No unbiased discovery. Time and reagent intensive.	Multiplexed visualization of CAF proteins (α-SMA, FAP, PDPN) with immune markers to define stromal-immune niches	https://www.quanterix.com/phenocycler-fusion-2-0/ accessed on 10 July 2026
Imaging Mass Cytometry	Subcellular resolution (1 µm)	Targeted proteomic	Yes	Mass-based, not fluorescence-based → avoids autofluorescence → well suited to dense desmoplastic stroma.	Same region cannot be used after imaging due to destructive acquisition of images. Slower acquisition	Quantitative protein-level mapping of ECM components (collagen, periostin) and CAF markers in autofluorescence-prone desmoplastic PDAC stroma	https://www.nature.com/articles/s41592-025-02889-8 accessed on 10 July 2026
Digital Spatial Profiling (GeoMx DSP)	Region based, not single-cell	RNA and protein multiomics. Whole transcriptomics available	Yes	Flexible, morphology-guided region of interest selection (myCAF sheath vs. tumor-distal stroma) Non-destructive image acquisition	No single cell resolution. Observer-dependent region of interest selection	Pre-treatment biopsy profiling for spatially resolved predictive biomarkers of therapy response (NOTUM, SERPINA3, CD8+ density)	https://brukerspatialbiology.com/products/geomx-digital-spatial-profiler/geomx-dsp-overview/ accessed on 10 July 2026

**Table 3:** Comparative overview of stromal heterogeneity, extracellular matrix (ECM) composition, and resistance mechanisms across upper gastrointestinal (GI) cancers.

Cancer Type	Dominant Stromal Characteristics	Principal CAF Populations	ECM Composition	Major Resistance Mechanisms	Spatial Evidence	Therapeutic Implications
Esophageal (Adenocarcinoma and squamous cell carcinoma)	CAF-rich stroma; desmoplasia starts at Barrett’s esophagus stage	iCAFs; myCAFs; mCAFs	Collagen, fibronectin, periostin, hyaluronic acid, tenascin C	Biophysical: Mechanical barriers to drug and immune cell entry; YAP/TAZ activation via mechanotransduction. Molecular: Paracrine signaling (HGF/MET, FGF/FGFR); exosomal miR-21 (STAT3 activation); PD-L1 upregulation.	Coordinated changes in ECM composition and tissue stiffness with progression from metaplasia, to dysplasia and adenocarcinoma; POSTN expression shifts from vascular to stromal cells during progression	ALK5 (TGF-β receptor I) inhibition (Vactosertib); selective RTK pathway inhibition (FGFR/MET).
Gastric	Variable desmoplasia: highest stiffness in diffuse type	myCAF1, myCAF2; iCAFs (in diffuse type); apCAFs; mCAFs; pCAFs	Collagen (types I and IV), periostin, fibronectin, hyaluronic acid	Biophysical: Increased matrix stiffness (CNN1+ CAFs) impeding drug and immune cells entry; YAP/TAZ activation via mechanotransduction. Molecular: Paracrine signaling (IL-6/STAT3, HGF/MET); metabolic reprogramming (reverse Warburg effect), Exosomal Annexin A6 (FAK-YAP activation); CXCL12/CXCR4 immune exclusion; gap junction communication (Cx43).	myCAFs (subtypes 1 and 2) form densely structured niches enveloping tumor nests; stroma-myeloid niches (THBS2+ CAFs and SPP1+ tumor-associated macrophages) mediate immune checkpoint resistance.	YAP/TAZ-TEAD inhibition (Verteporfin); C3-C3AR1 axis disruption; selective FAK or JAK2/STAT3 inhibition.
Pancreatic (PDAC)	Extreme desmoplasia, with stroma comprising the majority of tumor volume; highest tissue stiffness	myCAFs (along with PSCs dominating the tumor core); iCAFs; apCAFs;	Collagen (mainly type I), fibronectin, high levels of hyaluronic acid	Biophysical: High interstitial fluid pressure causing vascular collapse, preventing drug and immune cells entry; YAP/TAZ activation via mechanotransduction. Molecular: Metabolic symbiosis (glutamine transfer); ITGB1-mediated pro-survival signaling; T-cell exclusion by PSC-dominated core.	Desmoplastic core (dominated by activated PSCs) walls off tumor; myCAFs localize tumor-proximal while iCAFs are tumor-distal; compartmentalized subTMEs (deserted vs. reactive) correlated to chemoresponse.	ECM normalization (PEGPH20); FAK inhibition (Defactinib) to resensitize to immunotherapy; FAP-targeting CAR-T/ADCs; Angiotensin II blockers (Losartan); Collagenase nanomedicine.

**Table 4:** Active clinical trials targeting the stroma in upper gastrointestinal (GI) cancers.

Trial Number	Compound/Regimen	Mechanism	References
NCT06044311	Vactosertib + standard therapy	TGF-β receptor I (ALK5) inhibitor	[170]
NCT05669482	Defactinib + avutometinib + gemcitabine/nab-paclitaxel	FAK inhibitor + RAF/MEK clamp	[174,175]
NCT04106856	Losartan + chemotherapy/radiation	Angiotensin II receptor blocker, stromal decompression	[187]
NCT05547321	OMTX705 ± anti-PD-1 therapy	FAP-targeting antibody-drug conjugate	[158]
NCT03727880	Defactinib + pembrolizumab	FAK inhibitor + anti-PD-1	[173]
NCT04331041	Defactinib + adaptive SBRT	FAK inhibitor + radiation	[188]
NCT04543071	Motixafortide + cemiplimab + gemcitabine/nab-paclitaxel	CXCR4 antagonist (CXCL12/CXCR4 axis) + anti-PD-1	[189]
NCT04939610	[^177^Lu]Lu-FAP-2286 ± chemotherapy	FAP-targeted radioligand therapy	[190]

Abbreviations: FAK: focal adhesion kinase; FAP: fibroblast activation protein; PD-1: programmed death-1; SBRT: stereotactic body radiation therapy.

## Data Availability

The original contributions presented in this review are included in the article. Further inquiries can be directed to the senior author. All opinions expressed in this article are the personal views of the authors and do not represent the stance of the editorial team or the publisher.

## Abbreviations

The following abbreviations are used in this manuscript:

ADC	antibody-drug conjugate
AKT (Akt)	protein kinase B
ALK5	activin receptor-like kinase 5 (TGF-β receptor I)
apCAF	antigen-presenting cancer-associated fibroblast
CAF	cancer-associated fibroblast
CAR	chimeric antigen receptor
CNN1	calponin-1
CODEX	co-detection by indexing
COL1A1	collagen type I α1 chain
CPS	combined positive score
CTGF	connective tissue growth factor
Cx43	connexin 43
EAC	esophageal adenocarcinoma
ECM	extracellular matrix
EMT	epithelial-to-mesenchymal transition
EndMT	endothelial-to-mesenchymal transition
ENT1	equilibrative nucleoside transporter 1
ERK	extracellular signal-regulated kinase
EV	extracellular vesicle
FAK	focal adhesion kinase
FAP	fibroblast activation protein
FGF(R)	fibroblast growth factor (receptor)
GI	gastrointestinal
HA	hyaluronic acid
HER2	human epidermal growth factor receptor 2
HGF	hepatocyte growth factor
HIF-1	hypoxia-inducible factor-1
HR	hazard ratio
iCAF	inflammatory cancer-associated fibroblast
ICI	immune checkpoint inhibitor
IDO1	indoleamine 2,3-dioxygenase 1
IFP	interstitial fluid pressure
IL	interleukin
ITGB1	integrin β1
JAK	Janus kinase
LAT1	L-type amino acid transporter 1
LOX(L2)	lysyl oxidase (-like 2)
mCAF	matrix cancer-associated fibroblast
MERFISH	multiplexed error-robust fluorescence in situ hybridization
MET	mesenchymal–epithelial transition factor (c-Met)
MHC	major histocompatibility complex
MIBI	multiplexed ion beam imaging
MLC	myosin light chain
MMP	matrix metalloproteinase
MMT	mesothelial-to-mesenchymal transition
mTOR	mechanistic target of rapamycin
myCAF	myofibroblastic cancer-associated fibroblast
NF-κB	nuclear factor kappa B
OS	overall survival
pCAF	proliferative cancer-associated fibroblast
PDAC	pancreatic ductal adenocarcinoma
PD-1	programmed death 1
PD-L1	programmed death-ligand 1
PDGF	platelet-derived growth factor
PEGPH20	PEGylated recombinant human hyaluronidase
PDO	patient-derived organoid
PFS	progression-free survival
PLOD2	procollagen-lysine 2-oxoglutarate 5-dioxygenase 2 (lysyl hydroxylase 2)
POSTN	periostin
PSC	pancreatic stellate cell
ROCK1	Rho-associated protein kinase 1
RTK	receptor tyrosine kinase
SBRT	stereotactic body radiation therapy
scRNA-seq	single-cell RNA sequencing
SCC	squamous cell carcinoma
SDF-1	stromal cell-derived factor-1
SHG	second harmonic generation
α-SMA	α-smooth muscle actin
STAT3	signal transducer and activator of transcription 3
TAZ	transcriptional coactivator with PDZ-binding motif
TGF-β	transforming growth factor β
TKI	tyrosine kinase inhibitor
TME	tumor microenvironment
TNC	tenascin C
TNF	tumor necrosis factor
VEGF(R2)	vascular endothelial growth factor (receptor 2)
YAP	Yes-associated protein
